# CIN2 and Active Surveillance: Evidence from 48-Month Follow-Up in an HPV-Positive Cohort

**DOI:** 10.3390/cancers17233796

**Published:** 2025-11-27

**Authors:** Maria Teresa Bruno, Antonino Giovanni Cavallaro, Alessia Pagana, Roberta Siena, Giorgia Campo, Martina Somma, Alessandra Fiorito, Zaira Ruggeri, Gaetano Valenti

**Affiliations:** 1 Department of General Surgery and Medical Surgery Specialties, Gynecological Clinic, University of Catania, 95123 Catania, Italyroberta.siena1@studium.unict.it (R.S.);; 2 Department of Experimental and Clinical Medicine, University of Catanzaro, 88100 Catanzaro, Italy; 3 UO di Ginecologie e Ostetricia, ASP Siracusa, 96100 Siracusa, Italy; 4 Cervical Cancer Screening Unit, Level II, ASP Messina, 98123 Messina, Italy; 5 Gynecological Oncology, Humanitas, 95045 Catania, Italy

**Keywords:** CIN2, active surveillance, persistence CIN2, spontaneous regression, progression, risk-based management

## Abstract

Background: Conservative management of grade 2 cervical intraepithelial neoplasia (CIN2) is now supported by international guidelines, especially in young women or those seeking fertility. However, identifying subgroups at increased risk of progression remains a clinical priority, especially in settings with prolonged surveillance. Objectives: To evaluate the clinical evolution of CIN2 in an HPV-positive cohort followed under active surveillance for a maximum follow-up of 48 months, with particular attention to the risk of progression, persistence and regression, and associated predictors. Methods: Retrospective observational study conducted on a cohort of 237 HPV-positive women with a histological diagnosis of CIN2. Three possible clinical outcomes (progression, persistence, and regression) were analyzed. Patients underwent structured follow-up including cytology and colposcopy every 6 months, and HPV testing annually. Excisional treatment was performed only in case of progression or poor compliance. Logistic regression (univariate and multivariate) was applied to identify independent predictors of progression and regression. Results: After a maximum follow-up of 48 months. 61.3% of lesions regressed spontaneously, 13.1% persisted, and 25.5% progressed to CIN3. In multivariate analysis, high-grade cytology (ASC-H/HSIL) and HPV 16/18 infection were independently associated with a reduced likelihood of regression (OR ≈ 0.5, *p* < 0.01) and an increased risk of progression (OR ≈ 2.0, *p* < 0.05). Lesion size and age were not significant predictors. Importantly, no invasive carcinoma occurred during follow-up. Conclusions: Active surveillance for CIN2 remains a valid strategy in low-risk subgroups, but it is not universally safe. Data suggest that the combination of high-grade cytology and HPV 16/18 represents a high oncological risk profile, for which prompt treatment is indicated. Persistence beyond 36 months should be considered a cumulative risk marker, with implications for personalized follow-up and risk management. Further prospective, registry-linked studies are required to validate long-term safety and refine risk-based management strategies for CIN2.

## Simple Summary

The management of cervical intraepithelial neoplasia grade 2 (CIN2) remains debated, particularly in women of reproductive age. This study evaluates clinical outcomes over 48 months of follow-up in HPV-positive women with histologically confirmed CIN2 managed under active surveillance. Clinical outcomes—regression, persistence, and progression—were analyzed in relation to cytology, HPV genotype, and lesion size. Logistic regression models were used to identify predictors of disease course. After 48 months, 61.3% of lesions regressed, 13.1% persisted, and 25.5% progressed to CIN3. High-grade cytology (ASC-H/HSIL) and HPV 16/18 infection were significantly associated with a higher risk of progression (OR > 2, *p* < 0.01) and a lower likelihood of regression. Lesion size (1–2 vs. 3–4 quadrants) was not an independent predictor. CIN2 is a biologically heterogeneous lesion. While active surveillance appears safe in carefully selected low-risk patients, the presence of HPV 16/18 and high-grade cytology should prompt closer monitoring or early treatment. These findings are consistent with recent ESGO recommendations and the 2019 ASCCP risk-based management guidelines, supporting individualized, risk-based clinical strategies.

## 1. Introduction

In recent decades, the classification and management of cervical intraepithelial lesions (CIN) have undergone significant evolution, reflecting progress in understanding viral pathogenesis and the dynamics of neoplastic progression [1,2,3]. Major international scientific societies have periodically updated their recommendations with the aim of refining diagnosis, reducing overtreatment, and ensuring an optimal balance between oncological safety and reproductive health protection [4,5,6,7]. In this scenario, the lesion defined as CIN2 (Cervical Intraepithelial Neoplasia grade 2) has long occupied an ambiguous and complex position, at the crossroads between low- and high-risk forms [8,9].

CIN2 is characterized by considerable biological heterogeneity, not only in terms of histology, but also in terms of variability in clinical behavior [10]. Unlike CIN1, which is generally considered reversible and of little oncological relevance, and CIN3, which presents a substantial risk of progression to carcinoma in situ and invasive carcinoma, CIN2 represents a true “gray area” in management. Some of these lesions show an indolent course and can regress spontaneously, while others evolve into more serious forms, at times and in ways that are not always predictable.

Traditionally, the management of CIN2 has focused on early excisional surgery, using procedures such as LEEP (Loop Electrosurgical Excision Procedure), even in young patients or those planning to conceive. This practice was dictated by the need to prevent progression to more advanced forms, but in the absence of reliable tools to identify, upfront, cases truly at risk. However, a growing number of observational studies and meta-analyses have highlighted that spontaneous regression of CIN2 is far from rare, with estimated rates between 40% and 74%, significantly higher than those recorded for CIN3 (22–33%) [11,12,13,14,15,16].

In parallel, the burden of adverse effects associated with cervical surgery has become clear, especially during childbearing years. Excisional procedures such as LEEP have been associated with an increased risk of obstetric complications, including preterm birth, low birth weight, and cervical insufficiency, with an incidence proportional to the depth and volume of tissue removed [17,18]. These data have triggered a paradigm shift in the scientific community, promoting more conservative and risk-based management, particularly for young, HPV-positive, but immunocompetent women wishing to preserve fertility [19,20,21,22,23,24]. Furthermore, the recent population-based cohort study by Lycke et al. has further emphasized the obstetric implications of delayed excisional treatment in CIN2, reinforcing the need to identify women at increased risk who may not benefit from prolonged surveillance [25].

Recent consensus guidelines, including the 2019 ASCCP risk-based management system and the 2025 BSCCP/ESGO position paper [26], now support the option of active surveillance for biopsy-proven CIN2, provided that rigorous eligibility criteria and structured follow-up are maintained. In particular, the recently published BSCCP/ESGO position paper provides the most up-to-date European consensus on the management of CIN2 [26], has highlighted the feasibility and safety of active surveillance in selected women, while recommending excisional treatment after 24 months in cases of persistent CIN2. unless there is histological confirmation of regression to CIN1 or normal cytology. These recommendations converge with European frameworks such as NICE (2025), and GISCI (2024), promoting individualized management based on cytology, HPV genotype, colposcopic findings, and transformation zone visibility [6,7].

However, despite the growing acceptance of conservative strategies, important gaps remain in the literature, particularly concerning the long-term oncological risk of CIN2. This intermediate-grade lesion represents a biological “turning point,” where persistent infection with high-risk HPV genotypes (especially HPV 16 and 18) and concomitant high-grade cytology (HSIL/ASC-H) may silently progress toward CIN3 or carcinoma in situ. Recent population-based data by Lycke et al., linking active surveillance cohorts to national cancer registries, have shown that women managed conservatively for CIN2 exhibit a higher cumulative risk of invasive cervical cancer over 20 years compared with those undergoing immediate excision, particularly among women older than 30 years [27].

These findings underscore the importance of robust long-term follow-up data and emphasize the need for accurate risk stratification when selecting candidates for active surveillance, and to better define the predictors of regression and progression. In line with current European consensus recommendations, local excisional treatment should be offered after 24 months of active surveillance if CIN2 persists, unless histological regression to CIN1 or normal cytology is documented [26].

Given these considerations, evidence on the natural history of CIN2 beyond 24 months remains limited, especially in HPV-positive women managed in real-world clinical settings. In particular, consistent data on predictors of regression and progression and on the implications of prolonged persistence under modern risk frameworks are lacking.

The present study, therefore, aims to describe the clinical evolution of CIN2 in an HPV-positive cohort followed under active surveillance for up to 48 months, assessing rates of regression, persistence, and progression, and identifying key predictors associated with each trajectory.

Through a rigorous and integrated analysis of age, cytology, HPV genotyping, and lesion size, this study seeks to provide clinically useful elements for refined risk stratification, supporting safer and more individualized decision-making—avoiding unnecessary treatment in low-risk cases while preventing therapeutic delay in lesions with substantial progression potential. In an era in which personalized medicine is increasingly central to clinical practice, our work aims to offer practical risk-assessment tools that support balanced care pathways, combining oncologic safety with reproductive health preservation.

## 2. Materials and Methods

### 2.1. Study Design and Population

A multicenter, retrospective, observational study was conducted on a consecutive cohort of HPV-positive women with a histological diagnosis of grade 2 cervical intraepithelial neoplasia (CIN2), managed under active surveillance between 2016 and 2021. All histological slides were independently reviewed by two experienced gynecologic pathologists at the University of Catania, blinded to the clinical data. Only cases confirmed as CIN2 by both reviewers were included in the final analysis.

Primary cytology refers to the cytological result immediately preceding the diagnostic biopsy that confirmed CIN2, usually obtained within three months prior to the histological diagnosis.

Eligible women met the following inclusion criteria: age between 25 and 40 years; histological confirmation of CIN2 by two independent gynecologic pathologists; positivity for high-risk HPV genotypes (including HPV 16 and 18 as well as other high-risk types; abnormal cytology at baseline, fully visible transformation zone (TZ1 or TZ2) with complete visualization of the squamocolumnar junction (SCJ) and lesion margins and availability of regular follow-up for up to 48 months or until a defined clinical outcome (progression, regression, or persistence).

Exclusion criteria included: presence of glandular lesions (adenocarcinoma in situ or glandular dysplasia), previous cervical surgery (conization, LEEP, or hysterectomy), incomplete follow-up data, or early termination of surveillance (<12 months).

The final study population consisted of 274 HPV-positive women, with a mean age of 32.4 ± 5.1 years.

Information on HPV vaccination status was available for approximately 22% of women; none had received HPV vaccination prior to diagnosis.

Primary outcome: Histological regression of the lesion (CIN1 or no lesion) within 24 months.

Secondary outcomes: Long-term evolution up to 48 months: Progression to CIN3, CIN2, persistence rate. And identification of clinical and virological predictors (age, HPV genotype, lesion size).

### 2.2. Definition of Active Surveillance Protocol

Active surveillance was defined according to current European and international recommendations [26].

All women had baseline histological confirmation of CIN2; the follow-up protocol included: colposcopy and cytology every 6 months, HPV DNA testing annually, and repeat cervical biopsies in the event of cytologic or colposcopic worsening (transition from minor to major abnormalities according to the IFCPC classification).

In addition, a repeat biopsy was recommended in cases of persistent abnormalities or if colposcopic findings did not improve over time, and was performed at least once within the 12–24 month interval in accordance with clinical judgment.

At 24 months, a control biopsy was systematically performed for all women. If CIN2 persisted, excisional treatment (LEEP) was recommended, in line with European consensus guidelines.

However, a subset of patients (approximately 20%) after detailed counseling and written informed consent opted for extended surveillance beyond 24 months, mainly for fertility preservation, concomitant medical conditions, or for personal and work needs.

This decision was carefully documented in the clinical records and was based on the physician–patient discussion of the risks and benefits.

### 2.3. Clinical Follow-Up and Outcome Definition

The maximum duration of active surveillance was 48 months.

Clinical outcomes were classified as follows:

Regression: histologic downgrading to CIN1 or negative findings, confirmed by two consecutive negative co-tests (cytology + HPV) at 6-month intervals.

Persistence: unchanged CIN2 diagnosis at sequential histologic evaluation beyond 12 months.

Progression: histologic confirmation of CIN3 or adenocarcinoma in situ (AIS) at any point during follow-up.

Patients achieving regression were released from follow-up after confirmation of virological and cytological normalization; in selected cases, a confirmatory biopsy was performed.

Excisional treatment was immediately performed in cases of histologic progression, poor colposcopic visibility, or patient non-compliance.

Poor adherence was defined as missing at least two consecutive scheduled visits (colposcopy and cytology every 6 months) or absence from follow-up for more than 12 consecutive months.

### 2.4. HPV Testing and Genotyping

Combined ectocervical–endocervical samples were collected in ThinPrep medium for HPV DNA testing.

Viral genotyping was performed using the INNO-LiPA HPV Genotyping Extra II assay (Fujirebio Inc., Japan), capable of identifying 28 high-risk, low-risk, and undefined-risk genotypes.

High-risk HPV types included 16, 18, 26, 31, 33, 35, 39, 45, 51, 52, 53, 56, 58, 59, 66, 68, 73, and 82.

In this study, high-risk HPV included all oncogenic types detected by the INNO-LiPA assay; however, for analytical purposes, the subgroup carrying HPV16 or HPV18 was analyzed separately, given its well-established higher oncogenic potential.

In the case of multiple infections, the genotype was assigned according to the dominant high-risk mRNA positivity result.

### 2.5. Statistical Analysis

Statistical analysis was conducted using R software (version 4.3.1, R Foundation for Statistical Computing, Vienna, Austria), with the support of the dplyr, broom, ggplot2, flextable, and officer packages for data management, modeling, and results presentation. The main categorical variables (Cytology, HPV, Lesion) were binarized as follows: Cytology_bin: ASCUS/LSIL = 0; ASC-H/HSIL = 1; HPV_bin: no HPV 16/18 = 0; HPV 16/18 = 1; Lesion_bin: Lesion 1–2 = 0; Lesion 3–4 = 1. The primary outcome for the analysis was: Clinical Regression (1 = regression, 0 = no regression), Clinical Progression (1 = progression, 0 = regression; persistent cases were excluded). Univariate and multivariate binary logistic regression models were applied to identify independent predictors of CIN2 clinical regression, CIN2 clinical progression. For each model, the following were calculated: Odds Ratio (OR), 95% Confidence Intervals (95% CI), and *p*-value. Statistical significance was considered at *p* < 0.05. The ORs and their corresponding CIs were obtained using the exp(confint()) function, and the goodness of the model was assessed using the Akaike Information Criterion (AIC) and residual deviance.

### 2.6. Ethics Approval and Consent to Participate

This study conforms to the principles of the Declaration of Helsinki, as revised in 2013. The research was conducted through a retrospective review of medical records. In accordance with current legislation on observational studies (20 March 2008), the study protocol was submitted to the Catania 1 Ethics Committee of the Catania University Hospital. The committee did not request any modifications to the protocol and deemed informed consent unnecessary, as the study involved only retrospective analysis of anonymized clinical data.

## 3. Results

Of the 313 patients initially recruited into the study, 24 were excluded due to incomplete clinical data, and 15 due to poor adherence to follow-up. Therefore, the final sample analyzed consisted of 274 HPV-positive women with an initial histological diagnosis of CIN2, undergoing active surveillance for up to 48 months. The mean age was 32.4 ± 5.1 years (range 25–40). The median follow-up duration was 36 months (range 12–48).(Table 1)

### 3.1. Baseline Characteristics

The majority of women were nulliparous (56.9%), non-smokers (63.1%), and unvaccinated for HPV.

High-grade cytology (ASC-H/HSIL) was found in 48.2% of women, and HPV 16/18 infection in 46.0%.

Most lesions involved 1–2 quadrants of the cervix (60.6%), and the transformation zone was fully visible (TZ1–TZ2) in all cases, as per the inclusion criteria.

Ten women (3.6%) were immunocompromised due to systemic conditions or chronic corticosteroid therapy.

### 3.2. Overall Clinical Outcomes at 48 Months

During the observation period, three main clinical outcomes were identified: regression, persistence, and progression of CIN2. The most frequent outcome was spontaneous regression, observed in 168 patients (61.3%). In 36 women (13.1%), the lesion was persistent, while progression to CIN3 was documented in 70 cases (25.5%). Importantly, no patient developed invasive carcinoma during the 48-month observation period, confirming the reliability of active surveillance in the short to medium term, if well-selected (Table 2).

Analysis of clinical data highlighted several significant trends:

Regression was more frequent in younger women (25–30 years), who showed a regression rate of 64.3%, compared to 59.2% observed in the 31–40 age group.

Low-grade initial cytology (ASCUS/LSIL) was also associated with a higher probability of regression (70.4%), as was the presence of HPV genotypes other than 16/18, with a regression rate of 69.6%.

Lesion persistence affected 13.1% of the sample, but was concentrated primarily in patients with high-grade cytology (ASC-H/HSIL), HPV genotypes 16/18, and colposcopically extensive lesions (involving 3–4 quadrants).

Progression to CIN3 was more frequent among patients with ASC-H/HSIL cytology (32.6%) compared to those with low-grade cytology (19.0%).

Viral typing also showed a significant impact: among women positive for HPV 16/18, the progression rate was 32.5%, compared to 19.6% among patients with other viral genotypes, confirming the presence of a correlation between HPV 16/18 and an increased risk of progression.

### 3.3. Spontaneous Regression Analysis

To better understand which factors influence the likelihood of spontaneous regression of CIN2 lesions, both univariate and multivariate logistic regression analyses were performed.

#### 3.3.1. Univariate Analysis

Univariate analysis showed that high-grade cytology (ASC-H/HSIL) was associated with a significant reduction in the likelihood of regression, with an odds ratio (OR) of 0.44 (95% confidence interval: 0.26–0.71; *p* = 0.001). Similarly, HPV 16/18 genotype positivity was also inversely associated with regression, with an OR of 0.46 (95% CI: 0.28–0.75; *p* = 0.002) (Table 3).

In contrast, colposcopic lesion size (assessed as involvement of 3–4 quadrants versus 1–2) did not show a statistically significant correlation (*p* = 0.354), suggesting that macroscopic lesion size alone is not an independent predictor of regression.

#### 3.3.2. Multivariate Analysis

In the multivariate model, which simultaneously included cytology, viral genotype, and lesion size, high-grade cytology was confirmed as an independent factor associated with a lower likelihood of regression (OR = 0.50; 95% CI: 0.29–0.83; *p* = 0.008), as was HPV 16/18 genotype positivity (OR = 0.52; 95% CI: 0.31–0.86; *p* = 0.014). In this model, lesion size also did not reach the significance threshold (*p* = 0.80) (Table 4).

#### 3.3.3. Clinical Progression Analysis

Logistic analysis was also extended to assess factors associated with progression from CIN2 to CIN3. Again, the two variables significantly associated with an increased risk were: ASC-H/HSIL cytology, which more than doubled the risk of progression (OR = 2.38; 95% CI: 1.35–4.25; *p* = 0.003); and HPV 16/18 positivity, also associated with a doubled risk (OR = 2.28; 95% CI: 1.29–4.05; *p* = 0.005) (Table 5).

Initial colposcopic lesion size did not show a significant association (OR = 1.06; *p* = 0.851).

In the multivariate model, both high-grade cytology (OR = 2.17; 95% CI: 1.19–4.00; *p* = 0.012) and HPV 16/18 infection (OR = 1.96; 95% CI: 1.09–3.56; *p* = 0.025) remained significantly associated with progression. Lesion size (3–4 quadrants) also did not reach statistical significance (*p* = 0.372) (Table 6).

### 3.4. Summary of Results

In summary, data analysis suggests a consistent clinical profile among predictors of CIN2 regression and progression. Patients with high-grade cytology and HPV 16/18 infection have a significantly lower probability of spontaneous regression and, at the same time, a significantly higher risk of progression to CIN3. Macroscopic lesion size, although it may influence colposcopic appearance, was not a significant independent predictor in either model.

Importantly, age did not emerge as a significant predictor, a finding that likely reflects the narrow age distribution (25–40 years) and the predominant influence of viral and cytologic factors.

### 3.5. Temporal Distribution of Clinical Events

During follow-up, the timing of clinical events followed a consistent pattern.

The majority of regressions occurred within the first 24 months, while most progressions were observed between 24 and 36 months, with only minimal variation thereafter.

At 12 months, early regression was recorded in approximately 42% of cases, increasing to 57.7% at 24 months and reaching a cumulative 61.3% at 48 months.

Persistence was noted in 15.3% of women at 24 months and 13.1% at 48 months.

Progression to CIN3 occurred in 27.0% of patients at 24 months and 25.5% cumulatively at 48 months, indicating a plateau beyond the two-year mark.

These data demonstrate that the most dynamic phase of lesion evolution occurs within the first two years of observation, after which the clinical course tends to stabilize.

## 4. Discussion

The interpretation of CIN2 is subject to significant inter-observer variability, which may influence the apparent rates of regression or progression. Therefore, cytological and virological factors such as high-grade cytology and HPV 16/18 infection may act as diagnostic adjuncts rather than pure prognostic markers, helping to better classify biologically high-risk lesions.

In this study, we analyzed the long-term clinical outcomes of a cohort of 274 HPV-positive women with an initial histological diagnosis of CIN2, managed with an active surveillance protocol for up to 48 months. The results confirm the high biological heterogeneity of CIN2 lesions and highlight the importance of the combined assessment of clinical, cytological, and virological factors in predicting lesion progression.

The results of this study suggest that two variables—high-grade cytology (ASC-H/HSIL) and HPV 16/18 infection—are independent factors associated with both a higher likelihood of progression and a reduced likelihood of spontaneous regression.

These data are clinically significant when considered in the context of conservative management of CIN2, particularly in young women or those planning to have children, in whom immediate surgical treatment is avoided.

In our sample, regression occurred in 61.3% of cases, a result consistent with international literature, which estimates spontaneous regression of CIN2 in 50–70% of cases, particularly in women under 30 and in the absence of high-risk HPV infection [6,7]. These data suggest that the clinical profile associated with a favorable outcome includes younger age, the presence of intermediate-risk HPV strains, and a non-severe initial cytological picture.

The 48-month persistence rate (13.8%) is lower than other studies reporting 20–30% persistence rates at 24 months, suggesting the effectiveness of active surveillance in initial patient selection [24]. However, it should be considered that persistence beyond 36 months (36 patients in our study) has been associated in the literature with a significantly increased risk of progression to CIN3 or microinvasion [12,13,14].

In the present study, 25.5% of patients showed histological progression to CIN3 during follow-up. This figure appears slightly higher than that reported in similar studies, which indicate progression rates ranging between 10% and 20%. A possible explanation for this discrepancy lies in the composition of the cohort: in our sample, there was a higher prevalence of HPV 16/18 infections (40.2%) and ASC-H/HSIL cytology (38%) at baseline, two factors known for their oncogenic potential. This difference suggests that high-grade cytology at baseline is a potential indicator of evolutionary risk, as confirmed by previous studies [28].

Covariate analysis using logistic regression and Cox models confirmed that ASC-H/HSIL cytology and HPV 16/18 positivity were significantly associated with more than a doubled risk of progression (OR > 2, *p* < 0.01), while colposcopic lesion size (1–2 vs. 3–4 quadrants) did not show a statistically significant association, in line with what was reported by Kushwah, B et al. and in contrast with studies considering lesion size as a surrogate for viral load [29].

The study by Moscicki et al. also highlighted how HPV 16 infection is associated with a significantly slower regression, while other cohorts, such as that of the Arbyn group, indicated that HSIL cytology was the most robust predictor of progression, even more than isolated viral typing [11]. The ALTS trial emphasized the importance of stratification based on cytology and HPV [10].

Our study confirms these data and follows this trend, strengthening the hypothesis that the association between high-grade cytology and HPV 16/18 infection should be a criterion for exclusion from active surveillance. In these cases, active surveillance may delay appropriate treatment and expose patients to unnecessary oncologic risk; therefore, immediate excisional management should be preferred, as also supported by recent prospective evidence [12,23].

This distribution underscores the importance of the initial clinical profile in guiding prognosis. In particular, the combination of age > 30 years, HPV 16/18, and ASC-H/HSIL cytology appears to be associated with a significantly increased risk of progression to CIN3.

The 2019 ASCCP guidelines introduced a paradigm shift in the management of cervical pathology, moving from a logic centered on the single histological diagnosis to a decision-making model based on the cumulative risk of developing CIN3+.

In this context, clinical decisions (surveillance vs. treatment) no longer rely on a static classification but rather on an individual’s dynamic risk profile.

Accordingly, our multivariate logistic regression model—which identifies HPV 16/18 infection and ASC-H/HSIL cytology as independent predictors of progression—aligns with ASCCP recommendations, as it enables patient stratification based on risk profile.

This approach is clinically useful, helping to distinguish patients suitable for active surveillance from those requiring immediate treatment.

Such a perspective may help optimize clinical resources, reduce overtreatment in young women, and at the same time ensure adequate long-term protection for high-risk cases.

With a follow-up of up to 48 months, this study represents one of the few national studies documenting the medium-term clinical evolution of CIN2 in a selected cohort of HPV-positive women undergoing active surveillance.

During follow-up, 70 women showed clinical progression, while 36 had persistent lesions without signs of regression, for a total of 106 patients (approximately 45%) who did not experience spontaneous resolution of the disease. This finding is particularly relevant from an oncological perspective: even in the absence of progression to invasive cancer, persistence or prolonged progression represents a significant clinical and psychological burden, with potential future implications for cancer risk. In high-risk patients, conservative management could delay treatment of a lesion destined to progress, exposing these women to a higher risk of long-term invasive cancer.

In particular, persistence beyond 36–48 months is a well-documented risk factor for evolution towards CIN3 or microinvasive carcinoma, as already reported in the literature [2,12,14].

Our study confirms that, even in a context of rigorous active surveillance, a significant proportion of patients do not achieve spontaneous healing, especially in the presence of HPV 16/18 or HSI cytology. From this perspective, the data support the idea that active surveillance of CIN2 should be reserved for selected subgroups with low virological and cytological risk, while high-risk patients may benefit from earlier management. Furthermore, the extended 48-month follow-up, while allowing for a more robust assessment, also highlights how some patients maintain cytological and virological abnormalities for years, suggesting that the oncological risk is not static, but cumulative over time.

One of the main strengths of our work is the extended 48-month follow-up, which is longer than that of many studies in the literature, which limit themselves to 24–36 months. Persistence beyond 36–48 months represents a clinically relevant threshold, as it indicates ongoing viral activity and histologic stability that may precede progression to CIN3 or microinvasive carcinoma. Such long-standing persistence, even without progression, carries cumulative oncologic risk and justifies therapeutic intervention. The multicenter nature of the study could strengthen the generalizability of the findings to different clinical settings; the large number of cases and adherence to clinical guidelines make the data comparable with other settings. Furthermore, the availability of complete information on cytology, viral genotype, and lesion extent makes our dataset particularly robust for multivariate analyses.

However, our study has some limitations. First, the retrospective design, although useful for analyzing a large cohort, may introduce selection bias. Furthermore, we did not include behavioral or immune variables (e.g., smoking, contraceptive use, and immune status), which could influence the evolution of the lesions. A further limitation is the lack of centralized review of histological samples, which could introduce some inter-observer variability in the classification of lesions.

## 5. Conclusions

Active surveillance for CIN2 is a valid option in selected low-risk women, particularly those without HPV 16/18 infection or high-grade cytology, provided that strict follow-up is ensured. However, prolonged persistence beyond 36 months or the coexistence of HPV 16/18 and HSIL cytology should prompt early treatment, as these factors identify patients at high risk for progression. Future prospective studies integrating molecular and immunologic biomarkers could further refine risk stratification and improve individualized management of CIN2 [30,31,32].

## Figures and Tables

**Table 1:** Baseline demographic and clinical characteristics of the study population at 48 months.

Variable	*n* (%) or Mean ± SD	Regression *n* (%)	Persistence *n* (%)	Progression *n* (%)
Total women	274 (100)	168 (61.3)	36 (13.1)	70 (25.5)
Age (years)	32.4 ± 5.1	–	–	–
25–30 years	112 (40.9)	72 (64.3)	14 (12.5)	26 (23.2)
31–40 years	162 (59.1)	96 (59.2)	22 (13.6)	44 (27.2)
Parity	1.2 ± 0.7	–	–	–
Nulliparous	156 (56.9)	99 (63.5)	19 (12.2)	38 (24.3)
Parous	118 (43.1)	69 (58.5)	17 (14.4)	32 (27.1)
Smoking status	–	–	–	–
Non-smokers	173 (63.1)	111 (64.2)	20 (11.6)	42 (24.3)
Smokers	101 (36.9)	57 (56.4)	16 (15.8)	28 (27.7)
Hormonal contraception	–	–	–	–
Yes	72 (26.3)	45 (62.5)	9 (12.5)	18 (25.0)
No	202 (73.7)	123 (60.9)	27 (13.4)	52 (25.7)
Cytology at baseline	–	–	–	–
ASC-US/LSIL	142 (51.8)	100 (70.4)	15 (10.6)	27 (19.0)
ASC-H/HSIL	132 (48.2)	68 (51.5)	21 (15.9)	43 (32.6)
HPV genotype	–	–	–	–
HPV 16/18	126 (46.0)	65 (51.6)	20 (15.9)	41 (32.5)
Non-16/18 high-risk HPV	148 (54.0)	103 (69.6)	16 (10.8)	29 (19.6)
Lesion size (quadrants)	–	–	–	–
1–2 quadrants	166 (60.6)	100 (60.2)	16 (9.6)	24 (14.5)
3–4 quadrants	108 (39.4)	68 (63.0)	20 (18.5)	46 (42.6)
Immune status	–	–	–	–
Immunocompetent	264 (96.4)	163 (61.7)	35 (13.3)	66 (25.0)
Immunocompromised *	10 (3.6)	5 (50.0)	1 (10.0)	4 (40.0)
Past gynecologic history (endocervicitis, CIN1, HPV infection)	58 (21.2)	32 (55.2)	9 (15.5)	17 (29.3)

Note: Percentages and outcomes refer to 48-month follow-up. Median follow-up duration = 36 months (range 12–48 months). * An immunocompromised status is defined by HIV infection, autoimmune disease under immunosuppressive treatment, organ transplantation, chemotherapy, or chronic corticosteroid use.

**Table 2:** Clinical outcomes of the studied sample (48-month follow-up).

Variable	*n* Women	Regression *n* (%)	Persistence *n* (%)	Progression *n* (%)
Total	274	168 (61.3%)	36 (13.1%)	70 (25.5%)
Age 25–30 years	112	72 (64.3%)	14 (12.5%)	26 (23.2%)
Age 31–40 years	162	96 (59.3%)	22 (13.6%)	44 (27.2%)
ASC-US/LSIL cytology	142	100 (70.4%)	15 (10.6%)	27 (19.0%)
ASC-H/HSIL cytology	132	68 (51.5%)	21 (15.9%)	43 (32.6%)
HPV 16/18	126	65 (51.6%)	20 (15.9%)	41 (32.5%)
HPV non-16/18	148	103 (69.6%)	16 (10.8%)	29 (19.6%)
Lesion 1–2 quadrants	166	100 (60.2%)	16 (9.6%)	24 (14.5%)
Lesion 3–4 quadrants	108	68 (63.0%)	20 (18.5%)	46 (42.6%)

**Table 3:** Univariate logistic regressions at 48 months.

Variable	OR	95% CI	*p*-Value
Cytology	0.44	0.26–0.71	0.0011
HPV	0.46	0.28–0.75	0.0019
Lesion	1.27	0.77–2.12	0.35

Note: The odds ratio (OR) was calculated as exp (β). A *p*-value < 0.05 indicates statistical significance.

**Table 4:** Multivariate regression analysis for CIN2 regression at 48 months.

Variable	OR	CI 95%	*p*-Value
Cytology (ASC-H/HSIL vs. ASCUS)	0.50	0.29–0.83	0.0078
HPV (16/18 vs. others)	0.52	0.31–0.86	0.0116
Lesion (3–4 vs. 1–2 quadrants)	1.07	0.63–1.83	0.80

**Table 5:** Univariate logistic regression analysis for CIN2 progression.

Variable	OR	Lower 95% CI	Upper 95% CI	*p*-Value
Cytology_bin	2.38	1.35	4.25	0.003
HPV_bin	2.28	1.29	4.05	0.004
Lesion_bin	1.06	0.60	1.86	0.851

**Table 6:** Multivariate logistic regression analysis for CIN2 progression.

Variable	OR	Lower IC 95%	Upper IC 95%	*p*-Value
Cytology (HSIL)	2.17	1.19	4.00	0.012
HPV 16/18	1.96	1.09	3.56	0.025
Lesion 3–4	1.31	0.72	2.41	0.372

## Data Availability

The data are contained within the article.
